# Impact of Regional Green Development Strategy on Environmental Total Factor Productivity: Evidence from the Yangtze River Economic Belt, China

**DOI:** 10.3390/ijerph18052496

**Published:** 2021-03-03

**Authors:** Kailu Guo, Shixiang Li, Zhanqi Wang, Jianru Shi, Jun Bai, Jinhua Cheng

**Affiliations:** 1 School of Public Administration, China University of Geosciences, Wuhan 430074, China; guokl@tynu.edu.cn (K.G.); zhqwang@cug.edu.cn (Z.W.); j.r.shi@cug.edu.cn (J.S.); baijun_cug@cug.edu.cn (J.B.); 2 School of Management, Taiyuan Normal University, Jinzhong 030619, China; 3 Mineral Resources Strategy and Policy Research Center, China University of Geosciences, Wuhan 430074, China; chengjinhua100@126.com

**Keywords:** green development, environmental total factor productivity, policy effect, difference-in-differences, propensity score matching, Yangtze River Economic Belt

## Abstract

Chinese government policy officially identify the Yangtze River Economic Belt (YREB) as one of regional green development strategies firstly in 2014. This strategy can be regarded as quasi-natural experiment, this paper aims to test its impact on regional environmental total factor productivity (TFP). First, slack-based measure model is used to calculate the environmental TFP from 2005 to 2017 at provincial level. Second, based on Chinese official statistics, differences-in-differences (DID) method is applied to construct an evaluation model of policy effect, combining with the kernel matching in propensity score matching (PSM) method. The results show that environmental TFP of YREB has significant spatial differences, with characteristic of high-east and low-west, its average level is 11.69 percentage points higher than the national average. YREB strategy promotes regional economic growth, but it does no effect on the regional environmental TFP yet. Modelling suggests that YREB strategy may play a role in the short term. From the significance of the control variables, infrastructure construction level is positively correlated with environmental TFP, while per capita GDP, financial development and energy consumption intensity have negative effect on environmental TFP. Based on this, policymakers should focus on green development, promoting industrial transformation, and enhancing environmental protection.

## 1. Introduction

Nowadays, China’s economy has been transitioning from a phase of rapid growth to a stage of high-quality development. It is imperative that develop a modernized economy. As a matter of fact, like many countries in Asia and the Pacific, China still faces the prominent problem of balancing economic growth and environmental protection [1]. In 2018, data from the IEA, for every 1% increase in global economic output, carbon dioxide emissions increased by nearly 0.5%. Driven by the growth of energy demand, global energy related carbon dioxide emissions have increased by 1.7% [2]. For this reason, China has taken various measures for energy saving and emissions reduction, including command control tools (i.e., carbon emission intensity limit and quota limit) and environmental economic policies (e.g., carbon emissions trading system). It has become an inevitable choice in the critical period of China’s economic transformation to emphasize economic development on the premise of environmental protection. The greening of the economy can be a long-term driver of sustainable economic growth [3].

Yangtze River Economic Belt (YREB) is chosen as the object of this study because it is an important region for China’s green economic development. Relying on the Yangtze River’s advantages of convenient water transportation and nature resources, provinces in YREB have experienced large-scale development since 1990s. Eight provinces in the middle and upper reaches of the Yangtze River have vigorously developed the heavy chemical industry [4], their products are shipped to the coastal areas via the Yangtze River and exported abroad. According to National Bureau of Statistics of China (NBSC), from 1990 to 2018, steel production had increased from 15.49 to 195.41 million tons approximately. Chemical fertilizer outputs had also rocketed nearly fivefold from 7.87 to 38.34 million tons and cement production surged fifteen times. In addition, the number of enterprises with high energy consumption and high pollution is increasing rapidly, such as non-ferrous metallurgy, petrochemical, thermal power. However, with burgeoning economy, ecological environmental problems are becoming more and more acute. Over the last two decades, about 60% of China’s water pollution accidents have occurred in the Yangtze River basin. As well as the annual discharge of wastewater and chemical oxygen demand (COD) from the Yangtze River have accounted for more than 40% and 30% of the national total after 2010, respectively.

In this context, The State Council of China (SCC) issued the policy in 2014, namely Guiding Opinions on Golden Gramme to Promote the Development of the Yangtze River Economic Belt. For the first time, it defined the scope of the YREB, which contains the entire administrative area of 11 provinces (i.e., Shanghai, Jiangsu, Zhejiang, Anhui, Jiangxi, Hubei, Hunan, Chongqing, Sichuan, Guizhou, and Yunnan). The aim of this policy is to build YREB into a golden economic belt featuring more beautiful ecology, more smooth transport, more coordinated economy, more integrated market, and more scientific mechanisms. As developing green economy has gradually become the basic requirements of YREB. Consensus has been reached on ecological protection, but due to the different economic bases of each province, there are still some difficulties in the development options.

Combined with propensity score matching (PSM) and difference-in-differences (DID), this paper quantitatively analyzes the impact of YREB strategy on regional ecological efficiency. Considering the availability of data and the objectivity of research, on the basis of the statistics released by the Chinese government, the balance panel data is set up. Environmental total factor productivity (ETFP) is taken as an important indicator to measure the eco-efficiency. The contributions of this study are twofold: First, ETFP of 30 provinces in China are calculated, and the scores show that China’s provincial ETFP from 2005 to 2017 are on a downward trend and have a feature of high-east and low-west. Similarly, the YREB also have such characteristics. Second, in empirical part, the implementation of YREB strategy is regarded as the policy experiment, observing its impact on regional ETFP after 2014. The findings indicate YREB strategy had no positive effect on regional ETFP until the year of 2017, but it had effectively boosted regional GDP growth, especially for the middle reaches of the Yangtze River. The dynamic effect test shows that YREB strategy has hysteresis, in the long term, it can improve the regional eco-efficiency and economic growth. Meantime, energy saving and pollution emission reduction are still the main tasks of the 11 provinces in YREB. This study has important reference significance for evaluating the effect of national strategy on ecological environment protection and economic development and it also provides reference for policymakers.

The remainder of the paper is organized as follows: Section 2 is literature review; Section 3 introduces the materials and methods. Section 4 is the empirical results. Section 5 discusses the results further. Section 6 presents the conclusions and policy implications.

## 2. Literature Review

### 2.1. Roles and Functions of Regional Strategy

The implementation of macro policy by the government is one of the main means to promote the economic development in a specific region. This could mainly achieve through the regional development planning and a series of market regulation means. Like the desire to develop one’s region is strongly shared by voters, politicians and even some investors across the world. A simple definition of regional development is that efforts to enhance employment, income, wealth, and opportunity within a defined geographic area [5]. Market failures and external characteristics of public resource can justify government intervention. Governments will often attempt to implement policies, such as planning, financing, regulating, and managing [6,7,8]. Through the implementation of regional policies, the government tries to promote regional economy boom, social stabilization, and ecological environment [9]. However, it depends on many objective factors and conditions. For instance, because of the significant difference among the European Union regions, the Regional European Policy have both positive and negative effects on socio-economic development [10]. The development of regional economy can also lead to a more concentrated regional industrial structure and human capital agglomeration. Regional policies should adapt to this change and adjust according to market scale. Otherwise, there will be negative effects. Drucker and Feser found that the agglomeration economies were not an important mediating mechanism for the productivity effect associated with local industry structure [11]. Further, Yang and Pan posited that regional policy might not be effective in underdeveloped regions and could lead to misallocation of government fiscal resources [12]. In brief, regional policy is a response to public goods, externality, and market failure.

Existing literatures shown that the rapid economic growth of developing countries may lead to the deterioration of regional ecological environment [13]. Because of the public externality of the ecological environment, it is an effective means to protect the environment through policies, laws, and other national mandatory tools. Besides, technological innovation can improve eco-efficiency. Especially green technologies and innovation for the environment [14]. As environmental-related technologies positively contribute to the regional green growth, regional policy should encourage research and development. Econometric results outlined that regions-sectors characterized by higher levels of green technologies (GTs) are those facing better environmental performance [15]. And technological innovation of energy utilization is also important for regional environment. The traditional fossil energy, which once promoted industrial reform, is no longer sustainable. Non-renewable energy is detrimental to green growth in BRICS (i.e., China, India, Brazil, Russia, and South Africa) countries [16]. The governments should consider the energy-efficiency-increasing initiatives as potentially important instruments of regional development policy [17]. Due to the environmental pollution caused by burning fossil fuels, regional policy should also focus on restricting enterprises and encouraging them to make energy innovation and take more responsible of the society [18].

### 2.2. Green Economy and High-Quality Development

Neoclassical economic theory holds that economic growth mainly comes from labor force, capital, and total factor productivity (i.e., growth accounting). However, in the economic development that emphasizes quality, more factors need to be considered. For example, modernized economy emphasizes high-quality development, not only the speed of economy growth, but ecological environment, social welfare, and output efficiency [19]. When it comes to high-quality development, the structure of economy is gradually optimized, the new drivers of economy are taken place of the old ones, the development of the regional economic is coordinated, the regional ecosystem is more harmonious, and the living standards of the people is significantly improved [20]. Moreover, high-quality development can also provide the high-quality output of the whole society in an efficient way. Such as green growth policies could result in a virtuous shift towards high value-added sectors [21]. The local governance (e.g., renewable technologies or services) had been proved very effective in reducing emissions of particulate matter [22]. Normally, economic growth is thought to be an acceptable way of strengthen the social and environment sustainability for all nations [23].

More specifically, high-quality economy emphasizing green environmental protection has become the core of modern economic system [24,25]. Nevertheless, there are regional differences in economic development in many countries [26]. The implementation of regional policy was conducive to balancing this kind of difference, not only reinforced the internal integrity, but also made a region more attractive and competitive to the outside globalizing world [27]. It could be seen that industries and regional economies evolved as a result of the interplay between local and non-local factors [28].

### 2.3. Environmental Total Factor Productivity

The traditional way to measure the economic efficiency is total factor productivity (TFP), which has been researched extensively [29,30]. In models that emphasize TFP growth, national policies that improve capital and labor efficiency or change endogenous rates of technological change could promote productivity growth, thereby accelerating long-term economic growth [31]. Due to the implementation of environmental policies has a profound impact on productivity and economic growth rate, leading many countries to hesitate in formulating environmental policies and signing this kind of agreements. On one hand, the implementation of environmental policies will lead to the change of input factors (e.g., energy saving), this could lead to the decrease in output [32]. On the other hand, in the context of strong environmental policy constraints, the pace of technological innovation will accelerate. Improving technological innovation efficiency and management level will lead to output growth effects. These effects are related to productivity growth [33]. Based on this, the traditional productivity measurement techniques do not consider the impact of environmental pollution in production activities, resulting in the inability to distinguish the above effects. Owing to the increasing severity of environmental problems, the impact of the environment on all aspects of society has increasingly extensive. So that the measurement results of productivity without considering the environmental factor are often inaccurate [34].

Under the background that resources and environment are increasingly becoming the hard constraints of economic growth, compared with the traditional TFP index, it is more scientific to use environmental TFP (ETFP) to measure the growth quality of an economy. Therefore, scholars put forward the ETFP, which is considered the energy consumption and pollution emissions [35,36]. It can be regarded as a comprehensive indicator to measure the economic growth, ecological efficiency, and environmental sustainability [37,38,39].

### 2.4. Characteristics of Economic Region Based on River Basin

Many economically developed areas in the world are relying on river basin, for example, the Tennessee valley in United States, the Thames river in United Kingdom, and the Rhine in Germany. Due to the obvious characteristics of water resources in the river basin, this kind of areas generally face the problems of water resources utilization and water ecological protection [40]. As agriculture, environment, industry, and millions of households in the river basin depend on the water resource. With the economy and society booming, the ecological pressure it bears is also increasing [41]. Many scholars have carried out research on the ecological factors and economic ones in the river basin, but they cannot do without the discussion of water resources. And the same to the literatures about YREB. This strategical area is based on the Yangtze River Basin. It becoming a hot research area for Chinese scholars in recent years. Scholars have studied the society, economy, environment, and development of YREB from different perspectives. For example, articles about influencing factors (e.g., land, water resources, energy, labor, education, science, and technology, etc.) promoting the development of YREB [42,43,44]. Papers concern the main means of water ecological environment protection [45,46,47]. Research on regional economic differences and coordinated development of YREB [48,49,50].

However, throughout the above scholars, there is few empirical studies about YREB on whether the regional green development policy promote ETFP at provincial level, and it is also the main contribution of this paper. Combined with the characteristics of region water resource, chemical oxygen demand (COD) emission index in wastewater is considered in measuring ETFP. This paper provides a method reference for taking ETFP as the main indicator to measure regional eco-efficiency, as well as provide an empirical basis for deepening the YREB strategy.

## 3. Materials and Methods

### 3.1. Evaluation of ETFP

#### 3.1.1. SBM Model Considering Undesirable Output

This paper used the SBM (Slack-based measure) model considering the undesirable outputs, which was based on DEA (data envelopment analysis) method to evaluate the ETFP. In economic activities, people can get good output such as gross domestic product (GDP) after investing certain production factors, but at the same time, it also produces the bad output, such as wastewater, exhaust gas, and solid wastes, which people do not expect. According to Zhou et al. [51], carbon dioxide emission performance index selection ideas, considering a production process where each province employs capital stock (K), labor force (L), and energy consumption (E) as inputs to generate gross domestic product (Y) as desirable output and CO_2_ emissions and COD (U) as undesirable output. Usually, the production process, which includes the undesirable output of pollution emissions, is called environmental production technology. The production technology set can be defined as Equation (1):

(1) $$P\left(K,L,E\right)=\left\{\left(Y,U\right):\left(K,L,E;Y,U\right)\in P\right\}$$

Then, ETFP of 30 provinces in China from 2005 to 2017 were calculated by the SBM model of efficiency in DEA. The radial model assumes that all inputs or outputs vary in the same proportion, so it is considered unable to explain the possibility of excessive or insufficient output, i.e., the slack problem. In order to solve this problem, Tone [52] constructed a slack-based measure DEA, which directly added slack variables to objective function. The non-effective DMUs (Decision-Making-Unit) do not need to be improved in the same proportion according to the ray direction, this maximizes the improvement. On this basis, Tone [53] then proposed a non-parametric DEA scheme for measuring efficiency in the presence of undesirable outputs. As Equation (2) shows the measurement model, which considers the undesirable outputs:

(2) $$S\left(ETFP_{j,t}\right)=S_{0}\left(x_{0},y_{0},u_{0}\right)=\min\rho =\frac{1-\frac{1}{m}\sum_{i=1}^{m}\frac{s_{i}^{x}}{x_{i0}}}{1+\frac{1}{s+v}\left(\sum_{r=1}^{s}\frac{s_{r}^{y}}{y_{r0}}+\sum_{w=1}^{v}\frac{s_{w}^{u}}{u_{w0}}\right)}\;s.t.\left\{\begin{array}{c} x_{0}=X\lambda +S^{-};\\ y_{0}=Y\lambda -S^{y};\\ u_{o}=u\lambda +S^{u};\\ \lambda \geq 0,S^{-}\geq 0,\;S^{y}\geq 0,S^{u}\geq 0 \end{array}\;\;\;\;\;\;\;\;\;\;\;\;\;\;\;\;\;\;\;\;\;\;\;\;\;\;\;\;\;\;\;\;\;\;\;\;\;\;\;\;\;\;\;\;\;\;\;\;\;\;\;\;\;\;\;\;\;\right.$$

where $ETFP_{j,t}$ represents the ETFP of j province in t period and $S_{i}$, $S_{r},$ and $S_{w}$ represent the slack variable of input, desirable output, and undesirable output, respectively. ρ is a decreasing function, and ranges from 0 to 1. m, s, and v stand for the number of factors for inputs, desirable outputs, and undesirable outputs, respectively. If and only if ρ=1 and $S^{-}=S^{y}=S^{u}=0$, the DMU is SBM efficient. If ρ<1, the DMU is inefficient, so that inputs and outputs need to be improved.

#### 3.1.2. The Model of Malmquist Index

The Malmquist Index (MI) was first proposed by Malmquist [54]. Caves et al. [55] and Färe et al. [56] calculated the Malmquist index of DMU by geometric average method. Färe et al. [57] then further decomposed the Malmquist productivity index into technological progress and technical efficiency indices in order to analyze the drivers of productivity change [58]. This paper first calculated the efficiency levels of period t and period t+1 for each DMU using Equation (1). Then, the cross-efficiency of period t and period t+1 was evaluated. The MI is defined by the ratio of the above four efficiency values, it is expressed as Equation (3) from period t to t+1.

(3) $$M\left(x^{t+1},y^{t+1},x^{t},y^{t}\right)={\left[\frac{\theta_{0}^{t}\left(x_{0}^{t+1},y_{0}^{t+1}\right)}{\theta_{0}^{t}\left(x_{0}^{t},y_{0}^{t}\right)}\frac{\theta_{0}^{t+1}\left(x_{0}^{t+1},y_{0}^{t+1}\right)}{\theta_{0}^{t+1}\left(x_{0}^{t},y_{0}^{t}\right)}\right]}^{\frac{1}{2}}\;$$

where $x^{t}$ and $y^{t}$ stand for the values of DMU evaluated in period t. $x^{t+1}$ and $y^{t+1}$ stand for that in period t+1. $\theta_{0}^{t}\left(x_{0}^{t},y_{0}^{t}\right)$, which is equal to $S\left(ETFP_{j,t}\right)$ in Equation (1), is the efficiency of a DMU in t period. Whether input orientation or output orientation, if MI is greater than 1, it means productivity increases, and if MI is less than 1, it indicates reduced productivity.

### 3.2. Policy Effect Evaluation Model based on Unmeasurable Variables

In economics, it is often necessary to evaluate the policy effect. This kind of research is called program evaluation, and the program effect is also known as the treatment effect. All the participants were divided into treatment group or the treated. The control group or comparison group was composed of non-participants [59]. Rubin proposed a counterfactual framework, which was later called Rubin Causal Model [60]. It uses the dummy variable $D_{i}=\left\{0,1\right\}$ to indicate whether individual i participates in the project, where 1 is participating and 0 is not participating. $D_{i}$ is usually called the treatment variable. The average processing effect of project participants is called ATT (Average Treatment Effect on the Treated). In this paper, differences-in-differences (DID) and propensity score matching (PSM) method, proposed by Heckman et al., is used to estimate the policy effects [61,62]. This method is used when the treatment variable $D_{i}$ has unobservable variables that do not changed with the time. The advantage of this approach is that it can control the difference between groups, which is unobservable and time invariant. Supposing there are two periods of panel data, the period before or after the experiment (e.g., development policy) was $t_{0}$ or $t_{1}$. $y_{0t_{0}}$ is the potential outcome for all the participants, and the experiment has not yet occurred in period $t_{0}$. The experiment has already taken place in period $t_{1}$, so there are two potential outcomes, namely, $y_{1t_{1}}$ (the treatment group) and $y_{0t_{1}}$ (the control group).

The premise of PSM-DID is the assumption that the mean value can be ignored. Or to put it another way, if there is no experiment, the trend of variables in the treatment group and the control group is similar and have the same time trend. The assumption is $E\left(Y_{0it_{1}}-Y_{0it_{0}}|X_{i},D_{i}=1\right)=E\left(Y_{0it_{1}}-Y_{0it_{0}}|X_{i},D_{i}=0\right)$. If this assumption is established, the average treatment effect can be estimated based on matched samples. The Equation (4) is as follows:

(4) $$\hat{ATT}=\frac{1}{N_{1}}\underset{i\in C_{S}\cap \left\{i∶D_{i}=1\right\}}{\sum }\left[\left(Y_{1it_{1}}-Y_{0it_{0}}\right)-\underset{j\in C_{S}\cap \left\{j∶D_{j}=0\right\}}{\sum }w\left(i,j\right)\left(Y_{0jt_{1}}-Y_{0jt_{0}}\right)\right]$$

where $C_{S}$ is the common support, $\left\{i∶D_{i}=1\right\}$ is the treatment group, and $\left\{j∶D_{j}=0\right\}$ is the control group. $N_{1}$ is the effective number of individuals for the treated. $w\left(i,j\right)$ is the weight corresponding to the matched $\left(i,j\right)$. It can be determined by kernel matching.

Based on the above method, this paper sets the regression model as follows:

(5) $$Y_{it}^{PSM}=\alpha_{0}+\alpha_{1}province_{it}+\alpha_{3}province_{it}\times year_{it}+\sum \alpha_{j}x_{it}+\mu_{i}+\tau_{t}+\varepsilon_{it}\;\;\;$$

In Equation (5), Y is the explained variable. $province_{it}$, whose value is $\left\{0,1\right\}$, stands for individual-dummy variable, the value of 0 or 1 means the province is in the control group or in the treatment group. As well as, the value of $year_{it}$ of 0 or 1 indicate that it is before or after the time of the policy implementation, respectively. The coefficient $\alpha_{3}$, which indicates the net policy effect, is the core parameter that is to be estimated. $\alpha_{3}>0$ indicates that the policy has a positive effect. On the contrary, it is negative. $x_{it}$ is a series of control variables.$\varepsilon_{it}$ is a random error term. Furthermore, in this paper, Shanghai, Zhejiang, Jiangsu, Anhui, Hubei, Hunan, Jiangxi, Chongqing, Sichuan, Guizhou, and Yunnan are regarded as the treatment group, and the other 19 provinces in China are the control group. The implementation time of YREB strategy is 2014.

### 3.3. Data Sources

Based on integrity and accuracy, data of the period from 2005 to 2017 were collected for 30 provinces in China, excluding Tibet, Hong Kong, Macau, and Taiwan. Moreover, 2005 was set as the starting year because China formally has taken the energy efficiency and the carbon emission into the constraints of economic development. Since 2010, the Chinese government has begun to set specific targets for energy saving and emission reduction in the Twelfth Five-Year Plan for Social and Economic Development of the State and the Thirteenth one. Besides, as the official data of China’s total energy consumption is only up to 2017, considering it is an important indicator for calculating ETFP and CO_2_ (carbon dioxide) emissions, this paper took 2017 as the ending time. All the indexes related to prices were adjusted according to the unchanged prices in 2000.

However, the physical capital stock (K) and CO_2_ emissions need to be calculated. The physical capital stock of 30 provinces can be calculated by the perpetual inventory method [63]. The equation for estimating the level of capital stock is $K_{it}=K_{it-1}\left(1-\delta_{it}\right)+I_{it}$, where i represents the province, t stands for the year, δ is the capital depreciation rate, and I is gross capital formation. According to the existing literatures, most scholars take Chinese depreciation rate (δ) as 9.6%. The physical capital stock for the period from 2005 to 2017 can be estimated by the further equation: $K_{it}=\overset{t}{\underset{k=1}{\sum }}I_{ik}{\left(1-\delta_{ik}\right)}^{t-k}+K_{0}{\left(1-\delta_{i0}\right)}^{t}$, where $K_{0}$ is the China’s physical capital stock for the year of 2000, which was evaluated by Zhang [64].

In addition, according to the calculation method proposed by IPCC, CO_2_ emissions mainly comes from fossil energy combustion and cement production [65]. l represents seven fossil fuels (including coal, coke, gasoline, kerosene, fuel oil, diesel oil, and natural gas) that are considered, and carbon dioxide data for each province are calculated. For this, the equation is $C_{it}=\overset{7}{\underset{l}{\sum }}E_{itl}\times NCV_{l}\times CEF_{l}\times COF_{l}\times \left(44∕12\right)+Q_{l}\times EF_{cement}$, where E represents the total energy consumption of l in the year t of i province. The data for this was obtained from China Energy Statistical Yearbook from 2006 to 2018. NCV is average low calorific value of seven fossil fuels, CEF is carbon emission coefficient, COF stands for carbon oxidation factor, and 44 and 12 represents the molecular weights of carbon dioxide and carbon, respectively. Q stands for the cement output. $EF_{cement}$ is 0.527, which is the coefficient of CO_2_ emission in cement production [66].

Furthermore, the other variables used in this paper were mainly from China Statistical Yearbook, China Energy Statistics Yearbook, China Environmental Statistics Yearbook, China Industrial Statistics Yearbook, and the Statistical Yearbooks for 30 provinces over 2006 to 2018. The statistical characteristics of each variable are summarized in Table 1.

### 3.4. Variables Selection

Variables of models are shown in Table 2. As a comparison, GDP is taken as the explanatory variable in the policy effect evaluation of the YREB.

First, per capita GDP (PGDP), urbanization (Urb), service sector (SERVICE), ecological protection (EP), and regional characteristics (i.e., eastern, central, and western) were set as the covariates of propensity score matching. It is ensured that range of propensity score for the treatment group and the control group had the common support as far as possible.

Second, according to Equation (5), $\sum \alpha_{j}x_{\mathrm{it}}$ is a set of control variables that should be considered (see Table 2). Because the aim of YREB was economic development and ecological protection, the control variables of policy effect test were divided into two parts, i.e., the development variables and the protection ones. There were seven development variables used, including GDP per capita (PGDP), average years of education (EDUC), regional industrialization (INDUSTRY), financial development (FIN), foreign direct investment (FDI), innovation ability (PATENT and TVTM), infrastructure construction (ICL). Two protection variables were selected, i.e., regional energy consumption intensity (ECI) and regional pollution emission intensity (PEI).

## 4. Results

### 4.1. ETFP Calculation Results

**Figure 1:**
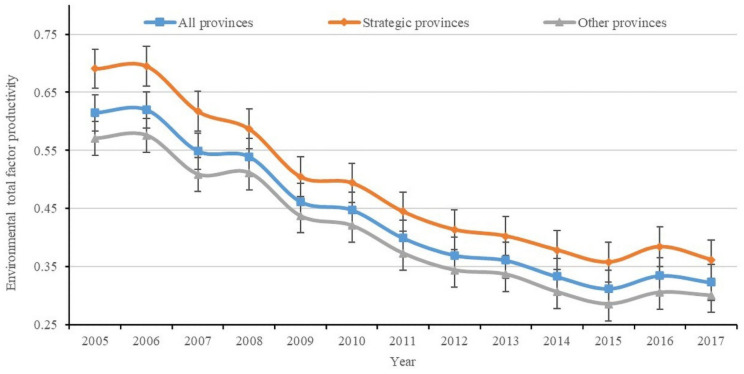
Mean value change curve of environmental total factor productivity (ETFP) in 2005–2017. Notes: The all provinces mean the 30 provinces in China in this paper. The strategic provinces represent the 11 provinces belong to Yangtze River Economic Belt (YREB), including Shanghai, Jiangsu, Zhejiang, Anhui, Jiangxi, Hubei, Hunan, Sichuan, Chongqing, Guizhou, and Yunnan. The other provinces are the other 19 provinces excluding YREB.

Figure 1 shows the ETFP’s average value of 30 provinces in China from 2005 to 2017, which is a downward trend during the past 13 years. The ETFP’s average value is increased in only a few years (2006 and 2016) compared with the previous year. The average ETFP of 11 provinces in the YREB (0.487) is higher than that of the whole country (0.436) and other provinces (0.406). This also means that the overall green economic efficiency of the YREB is better than the national average level.

**Figure 2:**
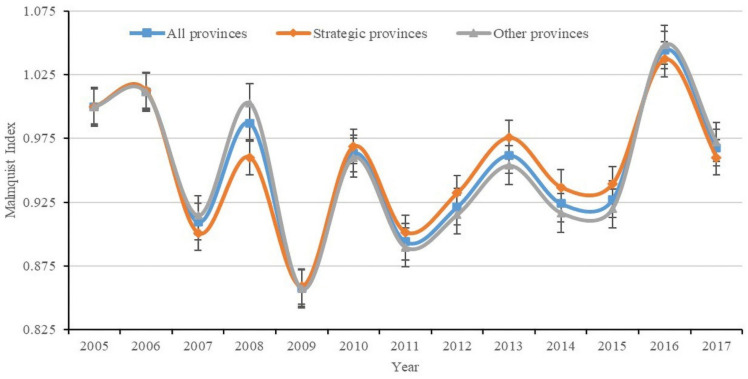
Mean value change curve of Malmquist Index (MI) in 2005–2017. Notes: The all provinces mean the 30 provinces in China in this paper. The Scheme 11 provinces belong to YREB, including Shanghai, Jiangsu, Zhejiang, Anhui, Jiangxi, Hubei, Hunan, Sichuan, Chongqing, Guizhou, and Yunnan. The other provinces are the other 19 provinces excluding YREB.

Figure 2 shows the average trend of Malmquist Index (MI), which represents the annual change value of ETFP. If the MI value is less than 1, it means that the ETFP has decreased compared with the previous year, otherwise, it represents an increase. MI of the strategic provinces and the whole sample unit increased only in 2006 and 2016, and the other years, during the period, are all less than 1. The MI of the other provinces, which do not belong to the YREB, are increased in 2006, 2008, and 2016. However, overall, the average value of MI in strategic provinces (0.9525) is higher than that of all provinces (0.9515) and other provinces (0.9509).

Table 3 shows the calculation results of ETFP and MI values for 30 provinces of 2005, 2010, 2015, and 2017. In general, the average value of eastern provinces is higher than that of central and western ones. This roughly reflects the reality of regional development differences in China. As far as the YREB is concerned, first, Shanghai, Jiangsu, and Zhejiang are in the eastern part of China, and they also belong to the lower reaches of the YREB. In 2017, the ETFP rankings of the three provinces were among the top five in China. Second, for the central provinces of YREB such as Anhui, Jiangxi, Hubei, and Hunan, their ETFP are all at the average level. However, Anhui decreased more obviously than the others over 2010 to 2017. Lastly, the four western provinces have the lowest level of ETFP. Among them, Sichuan ranks higher year by year, better than Chongqing, Guizhou, and Yunnan. Moreover, Beijing and Guangdong, which do not belong to YREB, are also among the top five in China. Qinghai, Ningxia, and Neimenggu are at the bottom of the country. They also belong to the Midwest.

### 4.2. Policy Effect Model Results

#### 4.2.1. Regression Results with Unmatched Samples

Table 4 shows the estimation results of the model for the policy effect on ETFP and on GDP with unmatched samples. There are four columns, two of them add the control variable, whereas the others do not. Results show that YREB policy has a positive effect on GDP growth. The net effect of policy increases regional GDP by about 6.01 percentage points. By contrast, the policy has no significant effect on ETFP. Due to the increase in $R^{2}$, the explanatory power of column (3) and column (4), which add the control variables, is stronger than that of column (1) and column (2). However, in terms of the significance for the coefficients of the explained variables, the impact of YREB policy on regional ETFP and GDP are both insignificant. In fact, the control variable has a certain role in the model. For instance, PGDP (lnpgdp), FIN (lnfin), ICL (lnicl), and ECI (lneci) have effects on regional ETFP, while PGDP (lnpgdp), FIN (lnfin), PATANT (lnpatent), TVTM (lntvtm), ICL (lnicl), and PEI (lnpei) play a role in regional GDP.

#### 4.2.2. Regression Results with Matched Samples

Since there are unknown factors out of control and systematic differences between the unmatched groups, the kernel matching method in the PSM model is used to eliminate the above influences. Before recalibrating the regression model with matched samples, they need to pass the balance test to confirm that the systematic differences are reduced. Many scholars indicated that a matching approach performed well in reducing imbalances between groups if the ASB (absolute standardized bias) is below 10% [67]. From Table 5, after matching the two groups, the balance test results show that the bias for the four covariates drop from −18.6%, −63.1%, 24.2%, −21.2% to 4.0%, 2.5%, 7.3%, 2.7%, respectively, which are all below 10%. This suggests that the matching comes out well.

Excluding the samples with no common support, the matched samples are used for regression. The results in Table 6 show that YREB policy only played a positive role in promoting regional GDP by 3.63%, but had no significant impact on ETFP as of 2014–2017. According to the column (3), the control variables lnpgdp, lnfin, lnicl, and lneci all have an influence on ETFP. However, both lnpgdp (−0.495) and lnfin (−0.254) are negative. These two indicators represent the level of economic development. The higher level of per capita GDP and financial development, the higher vitality of economic growth. However, due to national conditions, China is facing economic transformation period, the traditional “three-high” industries with high-pollution, high-emission, and high energy-consuming are still the main driving force for the regional economic growth. Therefore, the areas with high level of economic growth are also accompanied by serious environmental pollution, and YREB is exactly this area. Meanwhile, the calculation of ETFP includes energy consumption and pollutant emissions. So, there is the negative effect between these two economic indicators and ETFP in the empirical results. The coefficient of lneci is −1.0569, which is significant at the level of 1%. It means that the reduction in regional energy consumption intensity promotes the advance of regional ETFP. This further proves the above inference. The same evidence is shown in column (4), i.e., when the explanatory variable is regional GDP, the index’s coefficient of pollution emission intensity (lnpei) was 0.0247 at 1% level. All in all, the economic growth of YREB is accompanied by the increase in pollution emission intensity.

Besides above, the technological innovation (i.e., lnpatent and lntvtm) have no significant impact on ETFP, but they have an opposite effect on regional GDP. The sign of the two is opposite, which may be due to the interaction of other control variables. Generally, increasing technological innovation can effectively improve the regional ETFP [68]. The results of our tests, however, show that the scientific and technological innovation in the YREB has no significant effect on regional ETFP.

### 4.3. Dynamic Effect Test

It usually takes a long time for macro-policy to have certain effect. Nevertheless, the implementation of regional strategy is often affected by the response efficiency of local governments, the strength of policy implementation, and local interests [6]. However, we needed to find does the strategic impact of YREB exist? This requires further dynamic testing. The strategic interaction item of the YREB ($year_{it}\times province_{it}$) is lagged by 3 years. The results are shown in Table 7 that YREB policy has a significant positive impact on regional ETFP and regional GDP in the second lag period. In addition, according to column (2), it has a significant dynamic lag effect on regional GDP without adding control variables. This proves that our hypothesis exists.

### 4.4. Placebo Test

This paper takes the implementation of YREB strategy as a quasi-experiment. Placebo test is widely used in natural experiments. It can be used to test whether the implementation time and experimental grouping have an impact on the regression results. The specific measures are as follows.

First, the implementation time of the policy is set as 2010 randomly and the PSM-DID model is rerun. The results are shown in column (1) and (2) of Table 8 that there is no significant policy effect both on regional ETFP and regional GDP.

Second, the samples in the treatment group and the control group randomly are exchanged. The same number of provinces are selected from the eastern, central, and western regions. Beijing, Tianjin, Hebei, Shanxi, Henan, Neimenggu, Fujian, Guangxi, Shaanxi, Gansu, and Qinghai are set as treatment group. The other 19 provinces are designated as control groups. Then, model (7) is used to regress these samples. The results are shown in columns (3) and (4) of Table 8. Coefficient of strategic interaction of YREB is also not significant.

### 4.5. Regionalism Effect

The YREB is an economic development region based on the Yangtze River basin. Considering the differences of economic development level and ecological environment among different regions in this area, it was important to find that does YREB strategy have different influences on different regions? Therefore, this paper tests the policy effect on upstream, midstream, and downstream. As shown in Table 9, columns (1) and (2) are results of the downstream (Eastern). Columns (3), (4), (5), and (6) are results of the midstream (Central). Columns (7) and (8) are results of the upstream (Western).

The results show that YREB policy has a significant effect on the middle reaches but has little on the upper and lower reaches. In the model without control variables, YREB policy have a side effect on ETFP in the middle reaches that reduce it by 15.09%, but promote the growth of GDP by 13.7%. After considering control variables in column (5) and column (6), the regression results show that YREB policy only has a positive impact on the GDP of the middle reaches and the effect is weakened to 2.31%.

## 5. Discussion

The YREB Strategy as a macro-policy, which was issued by the Chinese government in 2014. Its purpose is to realize the regional ecology and economy common development. Through a streak of regional coordinated development policies and ecological protection policies, this strategy hopes to achieve regional common development and harmonious ecological environment. In view of the above empirical research, this paper focuses on the following discussion.

First, the YREB strategy has a clear implementation time, clear strategic intention, and detailed strategic planning. These above factors provide the basis for the empirical research. However, this paper has limits and difficulties of data collection. Some important indicators can only be collected until 2017, which leads to a small sample size in the model estimation, thus may influence the regression results. For instants, the regression results show that the YREB strategy has no significant effect on regional ETFP, however, in the dynamic test, the YREB policy has a lag effect in the long run, and it promotes regional ecological efficiency and economic growth. Actually, according to the latest indicators released by the National Bureau of Statistics of China [69], from 2017 to 2019, the energy consumption of GDP per 10,000 yuan in the YREB has decreased by 12.5%, and the turnover of technology market has increased by 128.6%, indicating that the industry in this region is to realize the transformation under the guidance of policies, with the concept of innovation-driven green development.

Second, in the rapidly developing countries, the economy can exceed sustainable limits, damaging the natural systems and vastly shrinking biocapacity [70]. The water resources in the basin have a huge attraction to enterprises, especially heavy chemical companies, and high energy consuming ones. The YREB is the fastest growing region in China, and the second industry is the support of its economic growth. The resources in the Yangtze river basin have caused more human activities and exacerbated regional ecological deterioration. Model regression results (see Table 6 and Table 9) also reflects the reality of problems, although the level of regional economic development has improved (e.g., per capita GDP and financial development), the ETFP is decreasing. In addition, that is why the strategy has a negative effect on ETFP in the middle reaches.

Third, based on this study, this paper proposes two possible research directions. For one, it is necessary to examine the policy effect on the main cities along the Yangtze River. This will be more microscopic and specific. It can also greatly increase the number of samples. For another, the aim of YREB is to build a golden economic belt featuring more beautiful ecology, more smooth transport, more coordinated economy, more integrated market, and more scientific mechanisms. The ecological environment protection of the YREB is the premise of economic growth, and seeking development through conservation is the key point. Therefore, it is also necessary to test whether the policies of development and protection in YREB have affected the regional ecological protection.

## 6. Conclusions and Policy Implications

### 6.1. Conclusions

The objective of this paper is to examine the impact of the YREB strategy on ETFP, which is used as an important index to measure the eco-efficiency. As a useful measurement index, it not only considers the ideal economic output but also considers the environmental impact of the bad output in the production process.

First of all, the resulting ETFP scores at the provincial level indicate the obvious spatial differences and the overall trend is decreasing. The variation of those scores across provincial regions provides an important insight for policy. The average ETFP for the eastern region in China is the highest (0.577), is followed by the central region (0.384), and is the lowest in the western region (0.327). As the YREB stretches across China’s territory from east to west, the ETFP of the YREB also accord with this characteristic, with high-east and low-west values. However, the average ETFP of YREB (0.487) is 11.69 percentage points higher than that of the national (0.436). This indicates that the YREB has a better efficiency advantage. Especially, the provinces in the downstream area, such as Shanghai (ranked first), Zhejiang (ranked fourth), and Jiangsu (ranked fifth). In recent years, although some provinces have declined (e.g., Anhui and Guizhou) obviously, the YREB’s provinces still have an advantage over others in their region.

Moreover, then from the empirical research, there are four conclusions. First, as a macro-policy, YREB does not have a significant impact on regional ETFP yet, it promotes regional GDP by 3.63%. Second, the policy effect tests for different regions in YREB show that there are obvious regional differences in the YREB. The downstream provinces have developed economy and high ecological efficiency. The economic growth rate of the middle reaches is the fastest. As the high-pollution and high-energy-consuming industries have greatly damaged the ecological environment, the ETFP of this area is generally mediocre. The development of the upstream area is backward, so the efficiencies are generally low. Third, according to the policy dynamic effect test, YREB strategy has a dynamic effect both on the regional ETFP and regional economic development, however, if the variables are lagged by 3 years, YREB strategy will be effective. Last but not least, from the significance of the control variables, the infrastructure construction level is positively correlated with ETFP, while per capita GDP, financial development and energy consumption intensity have a negative effect on ETFP.

### 6.2. Policy Implications

Modern human cultures have the technical, economic, and management capabilities to cope with natural resource and environment constraints and to hedge against risks and uncertainties. What is missing is an adequate political consensus to economically motivate these capabilities [71]. The above research conclusions provide the following implications for deepening the YREB strategy.

First, the promotion of regional ETFP is based on the joint influence of labor, capital, energy consumption, economic growth, and environmental pollution emissions. The key to coordinate these common elements lies in the transformation of development principle, the innovation of science and technology, the enhancement of human capital and the strengthening of environmental protection. Therefore, the YREB strategy should focus on improving the policy guidance in these aspects and bringing more policy dividend through greater regional policy.

Second, the successful experience of most developed economies in the world shows that there is no contradiction between economic development and environmental protection. Appropriate management system and coordination mechanism are important, as well as legal constraints. This paper shows that there are lots of differences in economic development and policy effects in YREB. Provinces should focus on exploring the system and mechanism of green economy development. They should break interest barrier and maximize common interests. In addition, the national legislative department should establish a complete system of laws to provide legal protection for protecting ecological environment.

Third, the governments in the YREB should encourage scientific and technological innovation in order to improve regional eco-efficiency. They should issue policies to attract high-end talents and expand the opening of the regional economy continuously.

Lastly, the governments should continue to deepen the transformation strategy of traditional industries, and promote enterprises in the region to take the road of green development. The whole system should strive for new three-high targets with the higher quality of supply system, the higher input–output efficiency, and the higher development stability.

## Figures and Tables

**Table 1:** The descriptive statistics.

Variable (Unit)	Obs.	Mean	S.D.	Minimum	Maximum
Labor (10^4^ person)	390	2609.16	1723.56	291.04	6766.86
Energy (10^4^ TCE)	390	12,975.22	8147.88	822.20	38,899.25
Capital (10^8^ yuan RMB)	390	33,306.63	28,406.38	1121.58	15,0512.20
${\mathrm{CO}}_{2}\;\mathrm{emissions}$ (10^4^ ton)	390	36,775.74	26,443.45	1473.234	13,6663.8
COD (10^4^ ton)	390	56.14	40.46	5.75	198.25
ETFP	390	0.436	0.210	0.120	1.041
GDP (10^8^ yuan RMB)	390	4499	3358	308.0	15,540
PGDP (yuan RMB)	390	10,360	5147	3309	27,922
EDUC (year)	390	8.723	1.000	6.378	12.66
Industry (ratio)	390	0.466	0.0814	0.190	0.615
FIN (ratio)	390	10.67	6.300	2.761	37.33
FDI (ratio)	390	2.406	1.875	0.0386	8.198
PATENT (pieces)	390	29,459	50,570	79	332,652
TVTM (ratio)	390	1.02	2.24	0.017	16.02
ICL (km/10^4^ person)	390	33.66	21.08	4.291	135.3
ECI (TCE/10^4^ yuan RMB)	390	3.49	1.791	1.244	10.95
PEI (ton/10^4^ yuan RMB)	390	36.81	18.51	1.829	77.38
EP (%)	390	9.15	6.10	1.50	30.30
URB (%)	390	52.96	13.96	26.87	89.60
SERVICE (%)	390	42.35	9.02	28.60	80.56

**Table 2:** Variable descriptions.

Type	Variable	Abbreviation	Description
Dependent variable	Environmental total factor productivity	ETFP	Calculate variable; see Equation (1)
	Gross domestic product	GDP	As a reference. Using 2000 price level to calculate real GDP over the years
Core explanatory variable	Province × year	–	Dummy variable (0,1)
Independent variable (development variable)	Per capita GDP	PGDP	Real GDP/regional total population
	Average years of Education	EDUC	Average years of educational attainment for people aged 6 years and above
	Industrialization	INDUSTRY	Regional secondary industry output value/GDP
	Financial development	FIN	Financial institutions’ balance of deposit and loan at year-end by region/GDP
	Foreign direct investment	FDI	Foreign direct investment/GDP
	Innovation ability ^a^	PATENT	Regional patents granted
	Innovation ability ^b^	TVTM	Transaction value in technical markets/GDP
	Infrastructure construction level	ICL	Length of regional highways/total regional population
	Urbanization	Urb	Share of urban population in the total population
	Service sector	SERVICE	Proportion of tertiary industry
Independent variable (protection variable)	Energy consumption intensity	ECI	Total consumption of energy/GDP
	Pollutant emission intensity	PEI	The sum of wastewater discharged, sulfur dioxide emission, and industrial solid wastes produced/GDP
	Ecological protection	EP	Area of nature reserves/total area of each province

Note: a and b represent the two indicators selected in this paper, which are used to refer to the innovation ability.

**Table 3:** Environmental total factor productivity (ETFP) calculation results of 30 provinces in China.

Region	Province	2005			2010			2015			2017			Means		
		ETFP	MI (%)	Rank	ETFP	MI (%)	Rank	ETFP	MI (%)	Rank	ETFP	MI (%)	Rank	ETFP	MI (%)	Rank
Eastern	Beijing	0.669	100.00	2	0.842	99.74	2	0.834	100.07	1	1.041	103.86	1	0.834	103.97	2
	Tianjin	0.771	100.00	26	0.464	90.57	7	0.288	92.90	13	0.299	100.50	12	0.462	93.24	10
	Hebei	0.520	100.00	9	0.404	99.05	16	0.247	90.64	21	0.256	92.89	21	0.373	94.91	17
	Liaoning	0.952	100.00	17	0.427	94.05	15	0.310	97.82	11	0.305	100.97	11	0.487	92.39	8
	Shanghai *	1.011	100.00	24	1.012	99.08	1 ↑	0.740	97.35	2 ↓	0.845	84.17	2	0.913	99.25	1
	Jiangsu *	0.710	100.00	15	0.571	100.50	5 ↑	0.440	96.45	5	0.464	100.27	5	0.552	96.86	5
	Zhejiang *	0.856	100.00	29	0.651	106.63	4 ↑	0.483	95.05	4	0.469	96.82	4	0.625	95.58	4
	Fujian	0.475	100.00	3	0.445	97.89	11	0.333	96.59	7	0.336	98.55	7	0.407	97.44	16
	Shandong	0.603	100.00	21	0.458	90.82	8	0.289	92.70	12	0.335	96.87	8	0.433	96.06	12
	Guangdong	1.020	100.00	5	0.737	98.56	3	0.497	95.72	3	0.487	96.18	3	0.712	94.74	3
	Hainan	1.018	100.00	8	0.547	100.93	6	0.331	90.25	8	0.339	96.11	6	0.551	92.46	6
Central	Anhui *	1.018	100.00	1	0.457	101.18	9 ↓	0.287	92.16	14 ↓	0.283	96.94	15 ↓	0.503	90.92	7
	Shanxi	0.355	100.00	22	0.314	98.28	25	0.195	94.14	26	0.227	114.53	23	0.284	96.91	25
	Jiangxi *	0.575	100.00	16	0.446	103.24	10 ↑	0.316	90.82	10	0.291	94.86	13 ↓	0.432	95.05	13
	Henan	0.517	100.00	10	0.390	93.49	19	0.249	92.52	20	0.275	97.67	16	0.367	95.47	19
	Hubei *	0.562	100.00	12	0.439	101.00	12	0.319	94.49	9 ↑	0.323	96.82	10 ↓	0.421	95.94	15
	Hunan *	0.610	100.00	13	0.434	100.44	14 ↓	0.334	93.52	6 ↑	0.330	95.98	9 ↓	0.441	95.54	11
	Neimenggu	0.312	100.00	18	0.232	93.56	30	0.137	89.66	30	0.120	84.57	30	0.217	93.12	30
	Jilin	0.528	100.00	14	0.344	94.96	23	0.259	94.22	18	0.274	93.92	17	0.360	95.28	20
	Heilongjiang	0.685	100.00	11	0.436	102.28	13	0.271	90.75	17	0.270	96.28	18	0.431	93.32	14
Western	Guangxi	0.495	100.00	6	0.329	95.78	24	0.252	94.79	19	0.227	89.70	22	0.343	94.34	24
	Chongqing *	0.509	100.00	30	0.375	93.86	21 ↑	0.274	93.54	16 ↑	0.266	95.81	20 ↓	0.370	95.24	18
	Sichuan *	0.421	100.00	25	0.365	101.28	22 ↑	0.277	94.11	15 ↑	0.287	101.41	14 ↑	0.349	97.19	22
	Guizhou *	0.320	100.00	7	0.290	101.92	27 ↓	0.222	93.28	24 ↑	0.203	96.76	25 ↓	0.273	96.65	26
	Yunnan *	1.005	100.00	28	0.396	68.48	18 ↑	0.243	92.59	23 ↓	0.223	96.37	24 ↓	0.478	89.59	9
	Shaanxi	0.463	100.00	23	0.381	95.85	20	0.245	88.83	22	0.268	100.10	19	0.358	96.07	21
	Gansu	0.300	100.00	4	0.303	103.31	26	0.198	86.54	25	0.200	100.31	26	0.266	97.08	27
	Qinghai	0.339	100.00	20	0.284	95.06	28	0.151	88.66	29	0.129	91.48	29	0.247	93.03	28
	Ningxia	0.302	100.00	19	0.259	96.59	29	0.156	88.57	28	0.140	93.50	28	0.236	94.48	29
	Xinjiang	0.519	100.00	27	0.403	111.38	17	0.194	83.13	27	0.182	99.07	27	0.349	92.51	23

Note: MI represents Malmquist Index. The means represent the average value of ETFP from 2005 to 2017. “↑” and “↓” show the rank change of Provinces in the YREB and * indicates that the province belongs to YREB. According to the Division of the East Central and Northeast China published by the National Bureau of Statistics (NBS) in 2011, this paper divides the 30 provinces into three regions: eastern, central, and western.

**Table 4:** Regression results with unmatched samples.

Explained Variables	(1)	(2)	(3)	(4)
	lnetfp	lngdp	lnetfp	lngdp
$year_{it}\times province_{it}$	0.0626	0.0601 **	0.0101	0.0025
	(0.9264)	(2.6963)	(0.1944)	(0.1967)
lnpgdp			−0.3828 *	0.7464 ***
			(−1.7803)	(5.8494)
lneduc			−0.4386	−0.0241
			(−0.7694)	(−0.2623)
lnindustry			−0.1685	−0.0019
			(−0.8000)	(−0.0244)
lnfin			−0.2684 ***	0.0402 *
			(−3.3077)	(1.7050)
lnfdi			−0.0447	−0.0054
			(−1.5957)	(−0.9245)
lnpatent			0.0354	0.0373 *
			(0.5193)	(2.0086)
lntvtm			0.0006	−0.0117 **
			(0.0316)	(−2.2877)
lnicl			0.1819 **	−0.0223 **
			(2.5395)	(−2.0701)
lnpei			−0.0322	0.0206 ***
			(−0.9647)	(2.7581)
lneci			−1.0297 ***	−0.0306
			(−3.7848)	(−0.4216)
Controls	No	No	Yes	Yes
Entity fixed effect	Yes	Yes	Yes	Yes
Time fixed effect	Yes	Yes	Yes	Yes
N	390	390	390	390
R−squared	0.4607	0.2016	0.8594	0.9316

Note: absolute t statistics in brackets. * *p* < 0.10, ** *p* < 0.05, *** *p* < 0.01.

**Table 5:** Balance test results.

Explained Variables	Unmatched			Matched		
	Treated	Untreated	Bias	Treated	Untreated	Bias
lnpgdp	9.098	9.179	−0.186 *	9.123	9.105	0.040
lnep	1.778	2.157	−0.631 ***	1.935	1.920	0.025
lnservice	3.755	3.711	0.242 **	3.730	3.717	0.073
lnurb	3.903	3.95	−0.212 **	3.913	3.906	0.027

Note: * *p* < 0.10, ** *p* < 0.05, *** *p* < 0.01.

**Table 6:** Regression results with matched samples.

Explained Variables	(1)	(2)	(3)	(4)
	lnetfp	lngdp	lnetfp	lngdp
province×year	0.0404	0.0363 *	−0.0017	−0.0031
	(0.5026)	(1.7729)	(−0.0281)	(−0.2371)
lnpgdp			−0.4969 **	0.8366 ***
			(−2.3593)	(8.2370)
lneduc			−0.4521	−0.0647
			(−0.6784)	(−0.6175)
lnindustry			−0.1055	−0.0331
			(−0.4899)	(−0.4128)
lnfin			−0.2538 ***	0.0260
			(−3.3720)	(0.9358)
lnfdi			−0.0476	−0.0110
			(−1.3445)	(−1.6679)
lnpatent			0.0633	0.0336 *
			(1.0260)	(1.7515)
lntvtm			−0.0114	−0.0121 **
			(−0.6013)	(−2.4197)
lnicl			0.1961 **	−0.0081
			(2.2964)	(−0.8151)
lnpei			−0.0415	0.0247 ***
			(−1.1968)	(3.5305)
lneci			−1.0569 ***	−0.0585
			(−3.6169)	(−0.7141)
Controls	No	No	Yes	Yes
Entity fixed effect	Yes	Yes	Yes	Yes
Time fixed effect	Yes	Yes	Yes	Yes
N	330	330	330	330
R−squared	0.4641	0.1839	0.8580	0.9361

Note: absolute t statistics in brackets. * *p* < 0.10, ** *p* < 0.05, *** *p* < 0.01.

**Table 7:** The results of the dynamic effect test of the Yangtze River Economic Belt (YREB) strategy.

Explained Variables	(1)	(2)	(3)	(4)
	lnetfp	lngdp	lnetfp	lngdp
$Year_{i\left(t-1\right)}\times province_{it}$	−0.0028	0.0163	−0.0216	−0.0038
	(−0.0788)	(1.3170)	(−0.7497)	(−0.5351)
$Year_{i\left(t-2\right)}\times province_{it}$	0.0468	0.0205 ***	0.0660 *	0.0077 *
	(1.2587)	(4.4460)	(2.0077)	(1.7647)
$Year_{i\left(t-3\right)}\times province_{it}$	−0.0320	0.0156 **	−0.0210	0.0064 *
	(−1.4119)	(2.6675)	(−0.9331)	(2.0502)
Controls	No	No	Yes	Yes
Entity fixed effect	Yes	Yes	Yes	Yes
Time fixed effect	Yes	Yes	Yes	Yes
N	253	253	253	253
R−squared	0.5076	0.0993	0.8850	0.9220

Note: absolute t statistics in brackets. * *p* < 0.10, ** *p* < 0.05, *** *p* < 0.01.

**Table 8:** Placebo test results.

Explained Variables	(1)	(2)	(3)	(4)
	lnetfp	lngdp	lnetfp	lngdp
Province×year	−0.0114	−0.0090	0.0635	−0.0031
	(−0.1404)	(−0.4867)	(1.3745)	(−0.2434)
Controls	Yes	Yes	Yes	Yes
Entity fixed effect	Yes	Yes	Yes	Yes
Time fixed effect	Yes	Yes	Yes	Yes
N	330	330	330	330
R−squared	0.8561	0.9364	0.8605	0.9361

Note: absolute t statistics in brackets.

**Table 9:** Test results of policy effect in different regions.

Region	Downstream Area		Middle Reaches				Upstream Area	
Explained Variables	(1)	(2)	(3)	(4)	(5)	(6)	(7)	(8)
	lnetfp	lngdp	lnetfp	lngdp	lnetfp	lngdp	lnetfp	lngdp
province×year	0.0628	0.0122	−0.1509 ***	0.1370 ***	−0.0014	0.0231 **	−0.1619	0.0141
	(1.0725)	(1.2538)	(−5.4961)	(10.0338)	(−0.0594)	(3.4852)	(−1.3007)	(1.1945)
Controls	Yes	Yes	No	No	Yes	Yes	Yes	Yes
Entity fixed effect	Yes	Yes	Yes	Yes	Yes	Yes	Yes	Yes
Time fixed effect	Yes	Yes	Yes	Yes	Yes	Yes	Yes	Yes
N	143	143	49	49	49	49	110	110
R−squared	0.8447	0.9227	0.5556	0.2994	0.9710	0.9978	0.8838	0.9898

Note: absolute t statistics in brackets. ** *p* < 0.05, *** *p* < 0.01.

## Data Availability

The data presented in this study are available on request from the corresponding author (lishixiang@cug.edu.cn).
